# Patient-Specific Cardiovascular Computational Modeling: Diversity of Personalization and Challenges

**DOI:** 10.1007/s12265-018-9792-2

**Published:** 2018-03-06

**Authors:** Richard A. Gray, Pras Pathmanathan

**Affiliations:** 1 Division of Biomedical Physics, Office of Science and Engineering Laboratories, Center for Devices and Radiological Health, Food and Drug Administration, Silver Spring, MD 20993 USA; 2 Silver Spring, USA

**Keywords:** Computer modeling, Patient-specific, Precision medicine

## Abstract

Patient-specific computer models have been developed representing a variety of aspects of the cardiovascular system spanning the disciplines of electrophysiology, electromechanics, solid mechanics, and fluid dynamics. These physiological mechanistic models predict macroscopic phenomena such as electrical impulse propagation and contraction throughout the entire heart as well as flow and pressure dynamics occurring in the ventricular chambers, aorta, and coronary arteries during each heartbeat. Such models have been used to study a variety of clinical scenarios including aortic aneurysms, coronary stenosis, cardiac valvular disease, left ventricular assist devices, cardiac resynchronization therapy, ablation therapy, and risk stratification. After decades of research, these models are beginning to be incorporated into clinical practice directly via marketed devices and indirectly by improving our understanding of the underlying mechanisms of health and disease within a clinical context.

## Introduction

Patient-specific cardiovascular modeling is quietly emerging from decades of academic research and is beginning to transition to impact clinical treatment; these efforts complement the prominent, and well-deserved, attention focused on precision medicine in the fields of genetics [1, 2], oncology [3, 4], tissue engineering [5], and pharmaceuticals [6, 7]. In this manuscript, we begin with a discussion of individualized therapy followed by a brief overview of patient-specific modeling, then present a few examples of clinical applications in the field of cardiovascular modeling, and conclude with a description of some of the main challenges. We restrict our scope to macroscopic (> 1 mm) personalized mechanistic models of cardiovascular dynamics. The clinical utilization of patient-specific modeling involves addressing two very complex approaches (individualized therapy and computer modeling), and the appropriate implementation(s) and evaluation(s) of these approaches remain largely unknown and a matter of ongoing discussion.

## Individualized Therapy

**Fig. 1:**
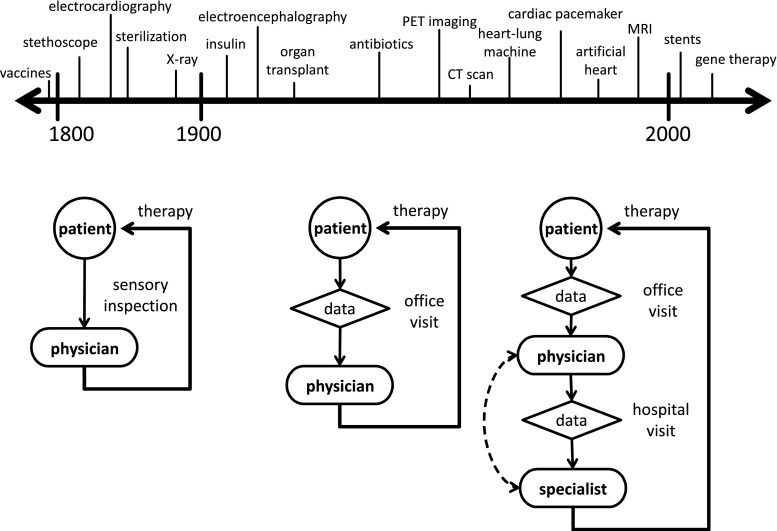
Evolution of medicine. (Top) selected timeline of advances in medical technology; (bottom) transformation of clinical practice. None of the times are meant to be interpreted precisely

The goals of medicine have always been patient-centric and include the relief of pain and suffering, curing of disease, and the promotion of health and prevention of illness. Advances in medicine during the twentieth century were unprecedented and resulted from multiple revolutions (e.g., technological, digital, genetic, information). The physician-patient relationship during the twentieth century tracked these scientific advances and transformed from a qualitative sensory inspection to an increasingly data-driven approach (see Fig. 1). Another important transition in the practice of medicine was from an “experienced-based” to an “evidence-based” approach [8]. Although its principles date back earlier, the first use of the phrase “evidence-based medicine” in the literature was in the early 1990s and was followed by an immediate “meteoric rise in popularity” [9]. Sackett et al. define evidence-based medicine as “the conscientious, explicit, and judicious use of current best evidence in making decisions about the care of individual patients” and its practice as “integrating individual clinical expertise with the best available external clinical evidence from systematic research” [10]. Currently, there is much discussion regarding the potential benefits of “precision medicine,” also referred to as “patient-specific medicine” [3], often colloquially described as providing the right treatment at the right time to the right patient.

**Fig. 2:**
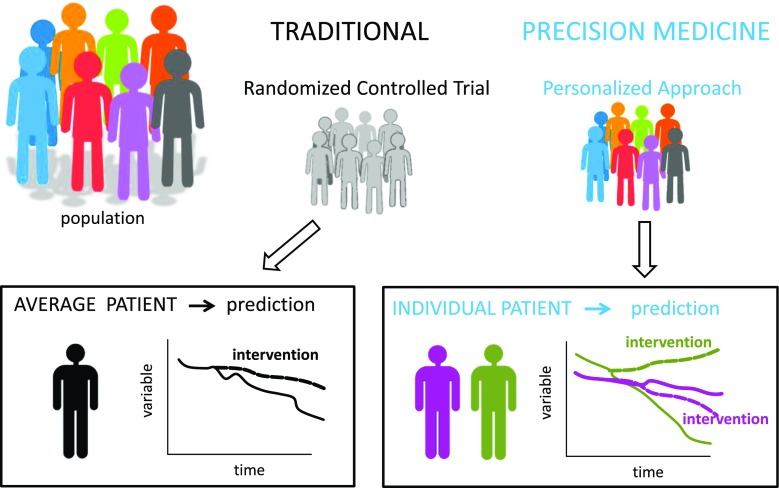
Precision medicine. Randomized controlled trials are the traditional approach for evaluating new medical therapies in which clinical advice is based on the predicted response of an “average” patient (black). Precision medicine offers an alternative approach in which it is envisioned that clinical advice is based on the predicted response of an “individual” patient; the responses of two different patients are displayed using purple and green (see text for details)

Precision medicine has significant appeal, in part because it is easy to envision significant benefits by transforming some clinical therapies from those intended for an “average patient” to those designed for an “individual patient;” Fig. 2 illustrates the potential benefit of such an approach. First, consider the traditional approach in which results from a randomized controlled trial (RCT) suggest a particular intervention is likely to benefit the “average patient” who meets the eligibility criteria of the RCT (see lower left panel of Fig. 2**)**. The intervention slows the progression of the disease (measured using some variable) as indicated by the dashed black line compared to no intervention (solid line), or standard of care, as determined in the control group from the RCT. Second, consider an alternative approach in which a clinical trial was conducted using a hypothetical patient-specific approach in which individual characteristics were accounted for in the study design, allowing for the prediction of an intervention for an “individual patient.” In this hypothetical case (see graphs in the lower right panel), the study results suggest that no intervention should be applied to patient 1 (purple) because it would have no benefit, but the intervention should be applied to patient 2 (green) who is predicted to benefit considerably from the intervention.

The implementation of patient-specific modeling will likely be varied with some approaches being similar to RCTs (e.g., adjusting inclusion/exclusion criteria) while others may be fundamentally different. Regardless of the particular implementation of patient-specific modeling, it seems prudent to compare and contrast the number of individuals a treatment is meant to benefit. At one extreme, a single treatment could be employed for the entire population, e.g., if you have a fever, take aspirin. This approach ignores all individual variability and encompasses a “one size fits all” attitude. At the other extreme, one can envision clinical treatment being tailored to a single individual; and if enough information was collected and understood, the “precision” treatment might include a combination of nutrition, exercise, and medicine to optimize that specific person’s health. Obviously, almost all conceivable medical interventions fall in between these two extremes. Accordingly, we view clinical approaches along a continuous spectrum of “personalization” within these extremes, and attempt to address the inherent tension between generalization and specification [11, 12]. In fact, the field of patient-specific cardiovascular modeling illustrates this concept nicely because one can accurately identify which aspects of the model are “personalized,” i.e., derived from the same patient for whom treatment is envisioned (see below for examples).

## Patient-Specific Cardiovascular Models

Cardiovascular modeling is a mature specialty spanning the disciplines of electrophysiological modeling, solid mechanics, electromechanics, and computational fluid dynamics. For each, the macroscopic structure-function relationships are represented by various partial differential equations based on conservation laws (i.e., conservation of charge, mass, momentum) [13]. Our review is meant to build upon previously published excellent reviews of cardiovascular modeling including whole heart electrophysiology [14] and electromechanics [15, 16], as well as fluid dynamics [17–20].

Patient-specific computational fluid dynamic models are being used to address aortic aneurysms [21], coronary stenosis [22], cardiac valves [19], and congenital heart disease [23, 24]. Bi-ventricular patient-specific models of electromechanics have been applied to heart failure [25–27], left ventricular assist devices [28], and cardiac resynchronization therapy [29–31]. Patient-specific models of electrophysiology have shown promise in regard to genetic mutations [32], ablation therapy [28], and clinical classification criteria [33].

**Fig. 3:**
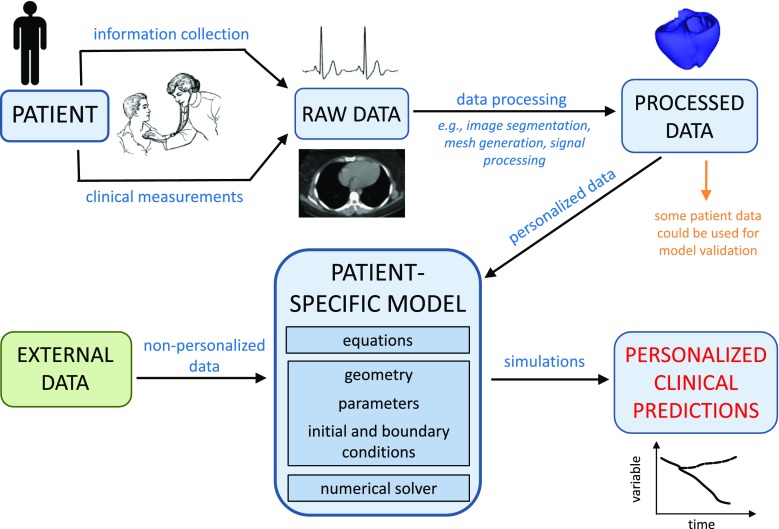
Patient-specific modeling workflow involves collecting and processing data from an individual patient and incorporating that data into a mathematical model represented digitally in a computer. The model incorporates the governing equations and parameters as well as mathematical representations of the patient’s geometry and boundary and initial conditions. Data collected from the patient can also be used for model validation (see the “Challenges” section for a discussion). Note that data used for model validation should be distinct to data used for model development

The construction of a patient-specific model typically involves a “workflow” (see Fig. 3) in which patient data is merged with equations and other “external data.” These pipelines involve obtaining information from the patient such as age, sex, survey results, and even physician diagnoses. In addition, measurements are taken from the patient using various instruments including sophisticated imaging modalities. Together, these streams represent the patient’s “raw data” which together with external data and equations governing the physical process being modeled are used to develop the patient-specific model. External data is any data that is not personalized, and can be obtained from a variety of sources such as experimental results, clinical studies, and the literature.

The amount and type of personalization in cardiovascular patient-specific models is quite varied, but almost always involves a geometrical representation of some part of the patient’s anatomy which is derived from a variety of clinical imaging modalities. These patient-specific geometrical representations are typically finite element “meshes” that are derived via the following steps: (1) imaging, (2) segmentation and reconstruction, and (3) interpolation and discretization (i.e., mesh generation) [18]. Other aspects of model personalization are quite diverse and necessarily discipline specific. Personalized parameters can be either directly measured from the patient or indirectly computed from patient data. For example, material properties such as stiffness or conductivity (i.e., electrical diffusivity) can be personalized, but estimation of personalized tissue material parameters can usually only be performed indirectly, unless biopsy samples are available. Model parameters need not be homogeneous; model developers must choose the amount (if any) of heterogeneity of model parameters, which also increases the opportunities for potential personalization. This choice should be well-justified because parameter heterogeneity increases model complexity which complicates model validation. Initial and boundary conditions for models can also be personalized. In some cases, this involves three-dimensional data which is difficult to acquire clinically and with much lower resolution compared to the final geometrical mesh. One- or two-dimensional clinical data can be converted to three-dimensions for model input using assumptions of symmetry; however, this approach may or may not be appropriate depending on the specific context of use for the model [34]. Below, we present the details of a few examples of patient-specific whole heart electromechanical models to illustrate the variety and complexity of model personalization.

Aguado-Sierra et al. [25] demonstrated the feasibility, and difficulty, of generating a patient-specific electromechanics model of the failing heart with a myocardial infarction and left bundle branch block in which anatomical representation, tissue stiffness, and electrical conductivity were all personalized. They generated a bi-ventricular mesh of a 65-year-old male from 2D echocardiographic recordings using a small number of manually identified landmarks. The infarct region was identified by an expert from an MRI stress test. Material stiffness parameters were estimated from the pressure (measured from the patient) volume (computed from the geometrical mesh) relationship. They used three-element “windkessel” lumped parameter models to represent the vasculature to establish boundary conditions and incorporated a non-personalized “cell” model (representing the detailed dynamics of the cardiac action potential) [35]. The regional electrical conductivity was personalized by reducing conductivity in the scar and elsewhere “to obtain an adequate activation sequence at the endocardium, and total activation time equal to the known QRS duration”. Similarly, Crozier et al. developed patient-specific models of bi-ventricular electromechanics from three heart failure patients in which regions of low conductivity were determined from non-contact mapping data [36]. In addition, passive tissue stiffness as well as windkessel and active tension parameters were personalized directly from the patient measurements [31].

The models described in the previous paragraph all include electrophysiology cell models that were *non-personalized*. A number of investigators have developed models by incorporating two-variable phenomenological cell models whose parameters were derived from patient data, into personalized geometries [37–41]. Phenomenological cell models are amenable to personalization because of their simplicity and the parameters are related to relatively easy-to-measure quantities (i.e., the rate dependence of action potential duration and conduction speed) [42–44]. However, the approach of using phenomenological models that do not represent the true action potential upstroke (e.g., Mitchell-Schaeffer [44] and Aliev-Panfilov [42] models) presents significant challenges [45]. Lombardo et al. fit parameters for both phenomenological and complex ionic cell models from patient data to derive personalized cell models which they incorporated into an atrial geometry and simulated reentry; they found that the results of the simulations for phenomenological and complex ionic cell models were similar for each patient, but spiral wave dynamics varied across patients [46]. Corrado et al. also generated personalized phenomenological cell models from patient data and simulated reentry in a two-dimensional sheet; they also found that spiral wave dynamics varied across the models developed for each patient [40].

Patient-specific modeling of cardiac electrophysiology, including simulation of cardiac arrhythmias, has begun to yield success in the clinical domain. For example, both Ashikaga et al. [47] and Arevalo et al. [48] generated personalized bi-ventricular geometries including regions of scar tissue and the surrounding “border zone” derived from magnetic resonance imaging (MRI) with late gadolinium enhancement. These workflows incorporated a non-personalized model of the electrophysiology of cardiac cells [35]. Ashikaga et al. demonstrated the feasibility of using such image-based personalized simulations to estimate ablation target sites for ventricular arrhythmias by simulating virtual arrhythmias in 13 patients and predicted sites for which ablating tissue within the arrhythmia circuit would terminate the arrhythmia [47]. Arevalo et al. constructed image-based personalized models of post-infarction hearts and simulated the propensity of 41 patient models to develop a virtual arrhythmia, and their predictions outperformed several existing clinical metrics in a retrospective analysis [48].

Notably, two medical devices have recently been marketed in the USA that include patient-specific cardiovascular models. Heartflow® FFRCT (fractional flow reserve derived from computed tomography) is a post-processing software for the clinical quantitative and qualitative analysis of image data for clinically stable symptomatic patients with coronary artery disease [49]. The workflow involves generating a personalized mathematical geometrical representation of the coronary arteries and performing computational fluid dynamics (CFD) simulations using lumped parameter models of the heart, systemic circulation, and smaller downstream coronary arteries as boundary conditions. Specifically, it provides FFRCT, a mathematically derived quantity, computed from simulated pressure, velocity, and blood flow information obtained from a 3D computer model generated from the patient’s static coronary CT images. The Medtronic CardioInsight® Cardiac Mapping System is a non-invasive mapping system for beat-by-beat, multichamber, 3D mapping of the heart [50]. This device includes solving the classic electrocardiographic “inverse problem,” i.e., computing the dipole sources on the heart surface from multiple potential measurements from the body surface [51]. This is accomplished by CardioInsight® via a workflow that involves computing the personalized torso and epicardial heart surfaces from CT images, and then computing the virtual electrograms on the heart surface using body surface potential signals recorded from > 200 electrodes from a vest worn by the patient [52].

Both Heartflow® FFRCT and CardioInsight® Cardiac Mapping System use CT images to generate a representation of the patient’s anatomy, and solve the governing equations using models with many non-personalized parameters. The personalized geometry alone for these devices provided unique clinical benefits (a scientific and objective measure of coronary blood flow and non-invasive cardiac mapping, respectively). Heartflow® FFRCT and CardioInsight® followed standard regulatory pathways for medical devices (the de novo and 510(k) pathways, respectively). The evaluation of medical devices by the FDA is unique to each submission; nevertheless, regulatory strategic priorities and ongoing activities indicate foresight in regard to patient-specific modeling. For example, one of the strategies for the Agency to the improve the effectiveness of the product development process is to “improve tools and approaches needed to catalyze the development of personalized medicine” [53] and one of the Center of Devices and Radiological Health’s (CDRH) regulatory science priorities for 2017 is to “develop computational modeling technologies to support regulatory decision-making” [54]. CDRH has published a Guidance on the reporting of computational modeling [55] and is involved in developing the American Society of Mechanical Engineering Standard entitled “Assessing Credibility of Computational Modeling and Simulation Results through Verification and Validation: Application to Medical Devices” [56]. CDRH also recently published a Guidance regarding evaluation of “software as a medical device” (SaMD), that is, software intended to be used for a medical purpose without being part of a hardware medical device, as opposed to a medical device which contains software [57]; this Guidance may cover some future patient-specific models. Finally, we note that computational modeling can play a key role in receiving FDA clearance or approval for medical devices. Faris and Shuren [58] state “For some devices, opportunities exist for leveraging alternative data sources, such as existing registries or modeling techniques, to allow regulators to have a good idea of the risks and benefits of the device without the need for conducting detailed trials.” As an example, Faris and Shuren [58] discuss the Medtronic Revo MRI ® pacemaker system, which was approved in 2011 as the first pacemaker indicated to allow patients implanted with the device to undergo magnetic resonance imaging (MRI) [58].

Given that heating would be most likely to occur in rare, worst-case conditions that would be difficult to predict clinically, relying on a clinical trial as the primary validation of safety would have required many thousands of participants. Instead, FDA approval rested primarily on robust mathematical modeling that was validated with bench studies and studies in animals. The modeling data, which simulated thousands of combinations of device and patient geometries and MRI scan conditions, provided strong evidence that even worstcase conditions would be very unlikely to result in detrimental lead heating.

It should be noted that the modeling referred to here was *not* patient specific, but we include the quote to demonstrate the role modeling, in general, can play in the regulatory process.

## Challenges

There are numerous far-reaching challenges to address for the wide-spread clinical utilization of patient-specific models. For example, how should clinical evidence be collected for development and evaluation such models? Here, we address the more limited challenges of the patient-specific cardiovascular models presented in this manuscript, which are typically developed according to the following steps: (1) define the problem to be addressed; (2) identify exactly how the model will be used, i.e., its context of use (COU); (3) select the model formulation including the governing equations; (4) decide upon boundary and initial conditions; (5) decide which aspects of the model will be personalized; (6) implement the model, i.e., construct the workflow; and (7) evaluate the predictive capability of the patient-specific simulations. There are challenges accompanying each of these steps. Perhaps, the biggest decision in step 3 is to decide if the model will be a multi-physics model (e.g., electromechanics or involve fluid-structure interaction) or not. We believe that one of the most challenging aspects of this process is to decide what level of detail to include in the model in step 3, which will depend heavily on the COU and the phenomena the model is meant to reproduce. For example, we believe that the scale and shape of resistive heterogeneities in electrical diffusivity that affect fibrillation [59] and defibrillation [60] dynamics are not well understood; thus, constructing patient-specific models to predict these phenomena would be problematic. For step 4, models that include solid or fluid dynamics tend to be more sensitive to boundary conditions and imposed constraints so they should be thoroughly explained and justified. We consider step 5 to be another of the most challenging stages, because the clinical settings impose unique constraints on what measurements can be made from the patient. These constraints on the level of personalization that are *possible* influence the ability to investigate the level of personalization that is* necessary *to achieve the required accuracy in model prediction. For example, if a patient-specific model which incorporates anatomical personalization has poor predictive capability, it can be difficult to determine if this is caused by a lack of material or functional personalization, or by other factors. However, technological advances continue to increase the amount of detailed and specific clinical information which will greatly aid the development of patient-specific models. Step 6 tends to be multifaceted and very complex involving advanced numerical methods including image processing, proper data filtering, and registration. The ideal workflow will be fully automated, to ensure reproducibility and remove user biases and errors.

One similarity of these macroscopic mechanistic cardiovascular models is that they are complex. There is an extraordinary amount of information required to fully understand these models (Fig. 3), and we recommend providing as much transparency as possible (perhaps in Supplementary Material for journal publications) regarding the relevant information such as the acquisition settings of the recording devices, signal/image/data processing, model assumptions, parameter values, and pre-processing stages such as computing unloaded reference geometry. A recent FDA Guidance on the reporting of computational modeling studies provides additional recommendations [55]. Numerous models have been developed over many years and understanding their details, rationale, and evolution becomes prohibitively difficult. Efforts to explain the scientific basis and clarify the rationale and assumptions of specific clinical modeling approaches [22, 52, 61] not only aid transparency but also improve the understanding and considering the advantages and limitations of the models. Similarly, presenting the history of model development, including errata, is helpful [62, 63]. Information regarding what part of the model is personalized, the workflow methodology, governing equations, model assumptions, and initial and boundary conditions are all important for evaluating patient-specific models.

Perhaps, the most significant challenge for patient-specific modeling is developing the appropriate methodology that both properly ensures patient safety and provides adequate evaluation (step 7). Here, we highlight a few examples of how certain activities could increase confidence in a patient-specific model. Testing accuracy and confirming assumptions at various stages along the workflows improves confidence in the process of model development as well as model robustness, though not necessarily the performance of the overall model. For example, the majority of workflows for generating bi-ventricular geometries for simulations of cardiac electrophysiology or electromechanics use imaging data to construct the heart wall boundaries, but use a variety of methods to generate the corresponding fiber fields [25, 31, 47]. Ideally, the fiber field for each patient would be acquired non-invasively using sub-millimeter diffusion tensor imaging (DTI) but this is not clinically feasible [64]. Vadakkumpadan et al. [65] assessed the accuracy of their fiber field estimation algorithm by comparing results to DTI data in six normal and three failing canine hearts. Confirmation of workflow methodology and assumptions using independent comparators provides valuable information, although spatial and temporal co-registration of data acquired from different recording devices is a complicating factor.

Parameter sensitivity analysis (quantifying the relative importance of changes in various input parameters on model output quantities) is extremely important because its helps refine model development and identify the parameters for which variability is significant, and helps to assess the robustness of model predictions. In fact, this process can be particular useful in the development of patient-specific models because it can help determine the level of detail to include in the model (including which parameters should be, or need not be, personalized). For example, Esptein et al. [66] studied the number of arterial 1D segments required to obtain adequate predictions of aortic flow. This is an important issue because the CFD models discussed in this paper all use 0D lumped parameter windkessel models as boundary conditions, and these simplifications do not account for wave propagation and reflection. Esptein et al. [66] systematically reduced 55 and 67 artery models by replacing a subset of segments with lumped parameter models to preserve the net resistance and compliance of the original model. They concluded that reduced models showed good agreement with the original models. In general, reduction of model complexity is advantageous because it simplifies model evaluation and increases computational efficiency, which may enable simulations to be feasible in clinically relevant timescales.

Validation of the patient-specific model, or more generally of the overall workflow, requires confirmation of the accuracy of predictions against clinical data. In the past, we have advocated [67] that the physiological modeling community takes advantage of the engineering field of verification, validation, and uncertainty quantification (VVUQ), which provides well-established methods for model assessment [68]. However, we also have described how the complexity and variability inherent to physiological systems introduce significant challenges to model validation; therefore, the relevance of engineering methods and best practices to general physiological modeling remains unclear, although efforts to develop similar methodology have begun [69–72]. Actually, VVUQ methods may be easier to apply to patient-specific models than more general models. One reason for this is that it is not clear what a general human model is supposed to represent (an “average” person? a “typical” person?), whereas for a patient-specific model, the model needs only to represent the patient from whom personalized data was obtained. Therefore, the process of performing model validation is conceptually simpler with a patient-specific model as compared to a generic human model. Moreover, patient-specific models decrease the challenges associated with uncertainty quantification (UQ). UQ involves quantifying the impact on model predictions caused by uncertainty in model parameters. Uncertainty in model parameters can originate from factors such as measurement error or physiological variability in the parameter. Using personalized parameter values removes the need to consider the potential impact, on predictions, of variability in (only) that parameter across individuals. For example, consider a hypothetical heart model that incorporates a generic non-personalized heart geometry, and whose simulations predict a clinical relevant quantity. Rigorous UQ requires evaluation of robustness of predictions given the range of heart shapes and sizes expected in the patient population. However, if the heart geometry is personalized, UQ only requires evaluation of robustness to heart geometry uncertainty arising from measurement (imaging) error and the mesh generation process.

On the other hand, patient-specific modeling introduces some subtleties in model validation that do not apply to generic models. Patient-specific modeling validation is typically carried out by testing the workflow in a clinical study cohort that is believed to be a representative of the indicated patient population, and if the study results are within acceptance criteria, the workflow is deemed sufficiently reliable for use in the greater patient population (e.g., supporting clinical evidence for Heartflow® and CardioInsight®). However, another distinct form of validation is also possible with patient-specific models. As illustrated in Fig. 3, one can envisage that every time the model is personalized to a new patient, some patient data is set aside to evaluate the model. This is somewhat analogous in machine learning to separating data into that used in the training phase and that used in the testing phase. For patient-specific models, it amounts to including validation as part of the overall workflow. For example, consider the following hypothetical workflow. Cardiac images and other data is used to generate a patient-specific heart model, which is then immediately tested by confirming that it successfully predicts that patient’s clinically measured pressure-volume loop. If the pressure-volume loop is successfully predicted, the model is used to predict a long-term clinical outcome; if not, the model is not used for this patient, or only used with caution. Regardless, the ability of this workflow to accurately predict the clinical outcome would still need to be evaluated using a clinical study. With this approach, however, confidence in predictions for any new patient would be supported by both the underlying clinical study results and the ability of the model to predict the new patient’s pressure-volume loop.

## Summary

The two FDA-cleared devices (Heartflow ® FFRCT and the Medtronic CardioInsight® Cardiac Mapping System) based on patient-specific cardiovascular modeling are based on well-established governing equations and conservation laws, but nevertheless represent a culmination of decades of basic research including the development of their workflows [65, 73–76]. The majority of previously published patient-specific cardiovascular models fall under the mission of the “Virtual Physiological Human” project which is to capture numerous and varied fragments of knowledge into predictive and personalized models that will make possible the investigation of the human body as a whole [77], although it should be appreciated that patient-specific cardiovascular models have long been used for surgical planning [23, 24]. The parameters and variables for these patient-specific models rarely have a direct correlate with clinically relevant quantities, and while this fact complicates the validation process, it provides unique opportunities to improve our understanding of the underlying mechanisms of health and disease within a clinical context [29, 31, 78].

The cardiovascular patient-specific models discussed here are inherently complex because of the difficulty in characterizing the underlying biology and their multiscale nature. As these models become more integrated in the clinical environment, we argue for the need for model transparency and robust evaluation frameworks that consider the risk to the patient and limitations in acquiring clinical data.
